# Increased blastomere number is associated with higher live birth rate in day 3 embryo transfer

**DOI:** 10.1186/s12884-022-04521-5

**Published:** 2022-03-11

**Authors:** Lifeng Tian, Leizhen Xia, Hongbo Liu, Yan Kou, Zhihui Huang, Xingwu Wu, Lu Fan, Jialyu Huang, Qiongfang Wu

**Affiliations:** 1 Center for Reproductive Medicine, Jiangxi Maternal and Child Health Hospital, Nanchang University School of Medicine, 318 Bayi Avenue, Nanchang, 330006 China; 2 School of Public Health, China Medical University, Shenyang, 110122 China

**Keywords:** Blastomere number, Live birth rate, Embryo transfer, In vitro fertilization

## Abstract

**Purpose:**

To study the relationship between blastomere number and pregnancy outcomes of day 3 embryo transfers.

**Methods:**

This retrospective cohort study included 2237 fresh single day 3 embryo transfer cycles from October 2013 to November 2020. Patients were divided into six groups according to the blastomere number on day 3: ≤ 6-cell (*n* = 100), 7-cell (*n* = 207), 8-cell (*n* = 1522), 9-cell (*n* = 187), 10-cell (*n* = 91) and ≥ 11-cell (*n* = 130). Generalized estimating equation analysis based on multivariate logistic regression model was performed to adjust for potential confounders.

**Results:**

The live birth rate (LBR) was 19.0%, 27.1%, 38.9%, 32.1%, 44.0% and 53.8% for the ≤ 6-cell, 7-cell, 8-cell, 9-cell, 10-cell and ≥ 11-cell groups, respectively (*P* < 0.001). Specifically, the ≤ 6-cell group was associated with reduced LBR compared with the 8-cell group (aOR 0.50, 95% CI 0.29–0.86; *P* = 0.013). Conversely, the odds of live birth were significantly increased in patients transferred with 10-cell embryos (aOR 1.62, 95% CI 1.03–2.53; *P* = 0.035) and ≥ 11-cell embryos (aOR 2.14, 95% CI 1.47–3.11; *P* < 0.001) when using the 8-cell embryo group as reference. Similar trends were also observed in the rates of positive hCG test and clinical pregnancy, while no significant differences were detected in miscarriage risk.

**Conclusion:**

Increased blastomere number was associated with higher LBR in fresh single day 3 embryo transfer cycles. This finding questions the consensus on the reduced developmental potential of fast-cleaving embryos. Further large prospective studies are warranted for confirmation.

**Supplementary Information:**

The online version contains supplementary material available at 10.1186/s12884-022-04521-5.

## Introduction

A major determinant of in vitro fertilization (IVF) success lies in the selection of embryos with high implantation potential. Various novel strategies have been proposed in recent years, including time-lapse imaging [1], preimplantation genetic testing for aneuploidy (PGT-A) [2], as well as analyses of follicular cell gene expression and culture media metabolite content [3, 4]. Nevertheless, morphological scoring of embryos at certain timepoints, advantaged by its simpleness and non-invasiveness, still remains to be the most widely applied method for quality assessment [5].

For cleavage-stage (day 3) embryos, fragmentation percentage, blastomere symmetry and cell number have been described as three most predictive characteristics [6, 7]. According to national data from the Society for Assisted Reproductive Technology Clinic Outcome Reporting System (SART CORS), live birth rate (LBR) was positively associated with increased cell number up to 8 but was reduced in embryos with over 8 cells [6]. Similarly, in the Istanbul consensus reached by European Society of Human Reproduction and Embryology (ESHRE)-Alpha scientists, it was claimed that an optimal day 3 embryo would have 8 equally sized blastomeres while embryos cleaving slower or faster are likely to be abnormal, thus leading to decreased implantation rates [7].

However, controversies on the developmental potential of fast-cleaving embryos have always existed from the 2000s. Compared with 8-cell embryos, some researchers found that fast-cleaving embryos on day 3 had similar or even significantly greater blastocyst formation and expansion rates [8–13]. Using array-based comparative genomic hybridization, Pons et al*.* [13] further demonstrated comparable ploidy between blastocysts derived from > 11-cell embryos and those from 8-cell embryos. These results bring into question current embryological dogma and recommended guidelines of scientific societies.

To date, only little research has focused on the LBR following fast-cleaving day 3 embryo transfer, which could be the primary endpoint in IVF treatment [6, 14, 15]. Hampered by small sample size and inadequate confounder adjustment, these studies also came up with inconsistent conclusions. Additionally, follow-up of neonatal outcomes has not been conducted, deserving further investigation for the safety of IVF offspring.

The main purpose of the present work was to evaluate the effect of increased blastomere number on pregnancy outcomes in a large cohort of patients undergoing fresh single day 3 embryo transfer cycles.

## Materials and methods

### Study design and patients

A retrospective study was carried out at the Center for Reproductive Medicine, Jiangxi Maternal and Child Health Hospital (JMCHH) affiliated with Nanchang University School of Medicine. All infertile women who had fresh single day 3 embryo transfers were screened from October 2013 to November 2020. Exclusion criteria included history of recurrent pregnancy loss, diagnosis of congenital uterine abnormalities, cycles using donor oocytes or PGT, as well as missing data in the electronic medical records. The study protocol was approved by the Ethics Committee of JMCHH (No. 2021–01) and was carried out in compliance with the Declaration of Helsinki. According to the Ethics Committee of JMCHH, informed consents were waived due to the retrospective nature, and all patients’ data were used anonymously.

### Controlled ovarian stimulation

The depot gonadotropin releasing hormone agonist protocol was used as previously described [16]. Briefly, patients were injected with 3.75 mg triptorelin acetate (Diphereline, Beaufour Ipsen, France) on day 2 of the menstrual cycle. If pituitary desensitization, as evidenced by endometrial thickness ≤ 5 mm, serum follicle-stimulating hormone (FSH) < 5 mIU/mL, luteinizing hormone (LH) < 5 mIU/mL and estradiol (E_2_) < 50 pg/mL, was achieved after 28 days, exogenous recombinant human FSH (Gonal-F, Merck Serono, Switzerland) was given to initiate stimulation. The starting dose was determined by the patient’s age, body mass index (BMI) and ovarian reserve, with subsequent adjustment made according to follicular response. When at least one follicle reached a mean diameter of 20 mm or two leading follicles were ≥ 18 mm, 250 μg recombinant human chorionic gonadotropin (hCG; Ovidrel, Merck Serono, Switzerland) was administered for ovulation triggering.

### Laboratory protocols

The oocytes were retrieved after 36–38 h via transvaginal ultrasound, and inseminated by conventional IVF or intracytoplasmic sperm injection (ICSI) according to semen quality. Fertilization check was performed at 17 ± 1 h post-insemination. The zygotes were subsequently transferred into pre-equilibrated sequential culture media (Vitrolife, Sweden), and incubated under mineral oil at a 37℃, 6% CO_2_ an 5% O_2_ condition. Day 3 embryos were assessed for cell number at 68 ± 1 h post-insemination, the standardized observation time-point recommended by ESHRE [7]. Other morphological characteristics were recorded simultaneously, including blastomere symmetry, fragmentation percentage and presence of multinucleation or vacuoles. During the study period, embryo scoring was conducted by the same team of four highly skilled embryologists, each with more than 5 years of experience. In order to reduce inter-observer variation, two embryologists were assigned for grading embryos derived from each patient.

### Embryo transfer

For patients undergoing fresh embryo transfer, endometrial secretory transformation was started on the day of oocyte retrieval with injected progesterone (60 mg/d; Xianju Pharma, China). Three days later, cleavage-stage embryo transfer was performed under the guidance of transabdominal ultrasound. Vaginal progesterone (P) gel (90 mg/d; Crinone, Merck Serono, Switzerland) and oral dydrogesterone (20 mg/d; Duphaston, Abbott Biologicals, USA) were used for luteal phase support, and continued until 10 weeks in cases of pregnancy confirmation.

### Outcome parameters

The primary endpoint of the study was LBR *per* transfer. Other outcome measures included the rates of positive hCG test, clinical pregnancy and miscarriage. Live birth was defined as the delivery of a viable infant beyond 28 weeks of gestation. Positive hCG test was defined as a serum β-hCG level of ≥ 5 IU/L at 12 days after transfer. Clinical pregnancy was identified by the detection of intrauterine gestational sac with fetal heart beat at 1 month after transfer. Miscarriage was defined as clinical pregnancy loss before the 24th gestational week, with early and late miscarriage classified as pregnancy loss at < 12 and 12–24 gestational weeks, respectively.

Obstetrical and neonatal outcomes were also collected from couples by specially trained nurses using standardized questionnaires. The telephone surveys were conducted during each trimester of pregnancy and after delivery. Main obstetrical complications included hypertensive disorders of pregnancy, gestational diabetes mellitus, intrahepatic cholestasis of pregnancy and placenta previa. Main perinatal outcomes included sex ratio, preterm birth (< 37 weeks), very preterm birth (< 32 weeks), post-term birth (≥ 42 weeks), low birthweight (< 2500 g), very low birthweight (< 1500 g), macrosomia (≥ 4000 g) and major congenital malformations.

### Statistical analysis

Patients were divided into six groups according to the cell number on day 3: ≤ 6-cell, 7-cell, 8-cell, 9-cell, 10-cell and ≥ 11-cell. The continuous variables were tested for normality using the Kolmogorov–Smirnov test. The data complying with normality were presented as means ± standard deviations and compared by one-way analysis of variance, while those that did not were expressed as medians (Q25, Q75) and compared by Kruskal–Wallis test. For categorical variables, data were expressed as the number and percentage of cases, with Pearson’s Chi-square test or Fisher’s exact test applied for comparison when appropriate. In addition, the 95% confidence intervals (CIs) of LBR were computed for each group by the normal approximation method.

Generalized estimating equation (GEE) analysis based on logistic regression model was performed to study the independent effect of blastomere number after controlling for potential confounders. The application of GEE mainly considered the clustered nature of data, as some patients contributed more than one cycle to the study [17]. The following variables were included: age, BMI, infertility duration, infertility type (primary or secondary), infertility diseases (tubal factor, male factor, ovulatory dysfunction, endometriosis, intrauterine adhesion or scarred uterus), IVF failure history (yes or no), total gonadotropin dose, endometrial thickness at trigger day, E_2_ level at trigger day, P level at trigger day, number of oocytes retrieved, fertilization method (IVF or ICSI), fragmentation percentage (< 10%, 10–20% or > 20%), blastomere symmetry (even or uneven), and presence of multinucleation/vacuoles (yes or no). Using the 8-cell embryo group as reference, we further calculated the crude and adjusted odds ratios (aORs) with 95% CIs of pregnancy outcomes for other categories. SAS 9.4 (SAS Institute, USA) was used for all statistical analyses. Two-tailed *P* < 0.05 was considered as statistically significant.

## Results

**Fig. 1:**
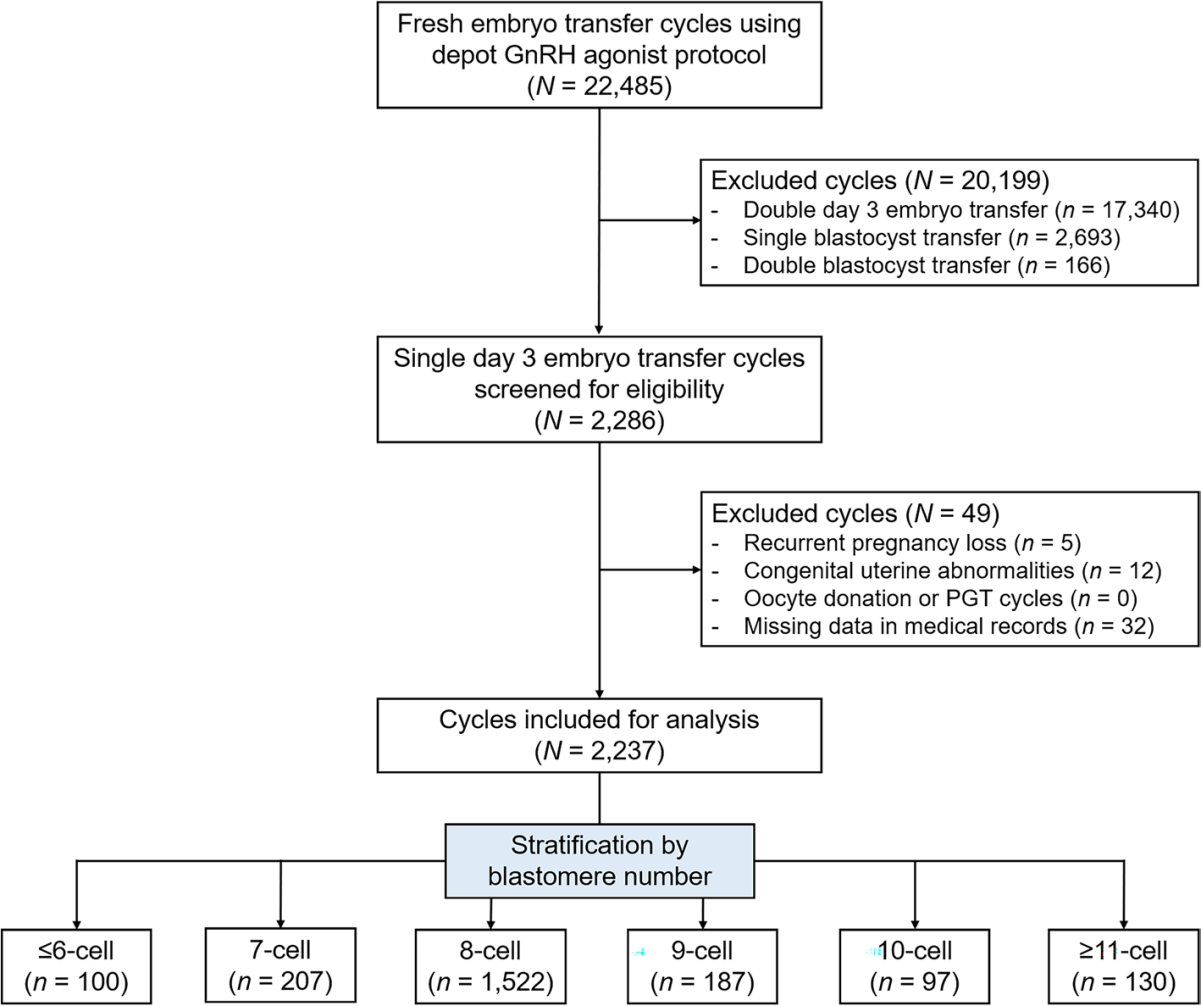
The flow diagram of the study. GnRH, gonadotropin releasing hormone; PGT, preimplantation genetic testing

The flow diagram of the study is shown in Fig. 1. During the study period, a total of 2286 single day 3 embryo transfer cycles were screened for eligibility, among which 2237 cycles from 2215 patients were included for further analyses. The number of cycles with ≤ 6-cell, 7-cell, 8-cell, 9-cell, 10-cell and ≥ 11-cell embryos was 100 (4.5%), 207 (9.3%), 1522 (68.0%), 187 (8.4%), 91 (4.1%) and 130 (5.8%), respectively. Baseline demographics of the study cohort are presented in Table 1. Briefly, the mean maternal age and BMI was 31.4 ± 4.7 years and 21.9 ± 3.4 kg/m^2^, respectively. Over 28% of the patients were nulliparous, and the primary disease of infertility was tubal factor (74.0%). With an average infertility duration of 4 years, 23.0% of women had a prior history of IVF failure.

**Table 1:** Baseline demographics grouped by blastomere number

	≤ 6-cell (*n* = 100)	7-cell (*n* = 207)	8-cell (*n* = 1522)	9-cell (*n* = 187)	10-cell (*n* = 91)	≥ 11-cell (*n* = 130)	*P*-value
Maternal age (years)	32.4 ± 4.9	31.9 ± 5.1	31.2 ± 4.5	31.3 ± 4.8	31.6 ± 5.1	31.3 ± 4.5	0.119
Maternal BMI (kg/m^2^)	21.9 ± 3.4	21.8 ± 3.0	21.9 ± 3.5	22.0 ± 3.3	21.6 ± 3.0	22.4 ± 3.4	0.639
Duration of infertility (years)	4.0 (2.0, 6.9)	4.0 (2.0, 7.0)	3.7 (2.0, 6.0)	3.8 (2.0, 5.1)	3.0 (2.0, 6.0)	4.0 (2.0, 6.0)	0.496
Secondary infertility, *n* (%)	62 (62.0)	130 (62.8)	1114 (73.2)	138 (73.8)	70 (76.9)	90 (69.2)	0.006
Infertility diseases							
Tubal factor, *n* (%)	71 (71.0)	137 (66.2)	1134 (74.5)	143 (76.5)	71 (78.0)	99 (76.2)	0.113
Male factor, *n* (%)	27 (27.0)	60 (29.0)	432 (28.4)	50 (26.7)	24 (26.4)	30 (23.1)	0.839
Ovulatory dysfunction, *n* (%)	8 (8.0)	31 (15.0)	156 (10.2)	31 (16.6)	9 (9.9)	21 (16.2)	0.017
Endometriosis, *n* (%)	16 (16.0)	14 (6.8)	113 (7.4)	12 (6.4)	7 (7.7)	7 (5.4)	0.040
Intrauterine adhesion, *n* (%)	3 (3.0)	14 (6.8)	90 (5.9)	13 (7.0)	7 (7.7)	9 (6.9)	0.742
Scarred uterus, *n* (%)	11 (11.0)	38 (18.4)	652 (42.8)	59 (31.6)	33 (36.3)	35 (26.9)	< 0.001
Basal endocrine profile							
FSH (mIU/mL)	6.5 (5.1, 8.2)	6.2 (5.1, 7.8)	6.2 (5.1, 7.5)	6.1 (5.3, 7.7)	6.5 (5.2, 7.8)	6.1 (4.9, 7.4)	0.886
LH (mIU/mL)	4.1 (2.8, 5.5)	3.8 (2.8, 5.3)	4.0 (2.9, 5.5)	4.0 (3.0, 5.6)	3.7 (2.7, 5.0)	4.2 (2.8, 5.5)	0.206
E_2_ (pg/mL)	42.5 (26.2, 64.5)	35.6 (26.0, 50.1)	35.9 (26.5, 49.0)	35.9 (26.1, 46.7)	34.9 (26.8, 49.0)	34.0 (25.0, 46.0)	0.252
PRL (ng/mL)	14.7 (9.9, 20.7)	14.5 (10.4, 19.9)	13.7 (10.1, 18.7)	13.4 (9.1, 18.1)	13.4 (10.7, 19.0)	13.9 (10.1, 19.7)	0.561
History of IVF failure, *n* (%)	31 (31.0)	58 (28.0)	326 (21.4)	47 (25.1)	26 (28.6)	26 (20.0)	0.043

Data are presented as mean ± standard deviation, median (Q25, Q75) or number (percentage)

*BMI* body mass index, *FSH* follicle-stimulating hormone, *LH* luteinizing hormone, *E*_*2*_ estradiol, *PRL* prolactin, *IVF *in vitro fertilization

Table 2 demonstrates the cycle characteristics and embryo parameters stratified by day 3 cell number. Comparison among the six groups revealed significant differences in total gonadotropin dose, peak E_2_ and P concentration, number of oocytes retrieved and fertilization method, while length of stimulation, endometrial thickness and LH level at trigger day were similar. In terms of embryo morphological features, the 8-cell group had a higher percentage of 10–20% fragmentation and lower proportion of uneven size than other categories (both *P* < 0.001). Contrarily, the presence of multinucleation or vacuoles did not differ significantly across groups (*P* = 0.889).

**Table 2:** Cycle characteristics and embryo parameters grouped by blastomere number

	≤ 6-cell (*n* = 100)	7-cell (*n* = 207)	8-cell (*n* = 1522)	9-cell (*n* = 187)	10-cell (*n* = 91)	≥ 11-cell (*n* = 130)	*P*-value
***Cycle characteristics***							
Duration of stimulation (days)	11 (9, 13)	11 (10, 13)	11 (10, 13)	11 (10, 12)	11 (10, 12)	11 (10, 12)	0.497
Total gonadotropin dose (IU)	2700 (2025, 3713)	2675 (1875, 3525)	2250 (1650, 3000)	2400 (1575, 3300)	2475 (1732, 3225)	2175 (1650, 2850)	< 0.001
LH level at trigger day (mIU/mL)	0.8 (0.6, 1.5)	0.8 (0.6, 1.3)	0.9 (0.6, 1.3)	0.9 (0.6, 1.3)	0.9 (0.6, 1.4)	0.9 (0.6, 1.3)	0.985
E_2_ level at trigger day (pg/mL)	1087.5 (663.1, 1859.0)	1360.0 (798.0, 1985.0)	1637.8 (1070.0, 2329.0)	1360.0 (818.0, 2134.0)	1374.4 (849.7, 2126.0)	1408.2 (950.7, 2086.4)	< 0.001
P level at trigger day (ng/mL)	0.6 (0.3, 0.8)	0.6 (0.4, 0.9)	0.6 (0.4, 0.9)	0.5 (0.3, 0.8)	0.6 (0.4, 0.9)	0.6 (0.4, 0.8)	0.009
Endometrial thickness at trigger day (mm)	10.3 (9.0, 12.2)	10.4 (8.7, 12.2)	10.6 (9.0, 12.3)	10.3 (8.8, 12.2)	10.2 (8.5, 11.6)	11.0 (9.0, 12.9)	0.130
No. of oocytes retrieved	7 (4, 8)	7 (4, 10)	10 (7, 13)	8 (5, 12)	8 (5, 11)	9 (6,12)	< 0.001
Fertilization method, *n* (%)							0.005
IVF	74 (74.0)	138 (66.7)	1194 (78.4)	143 (76.5)	71 (78.0)	107 (82.3)	
ICSI	26 (26.0)	69 (33.3)	329 (21.6)	44 (23.5)	20 (22.0)	23 (17.7)	
***Embryo parameters***							
Fragmentation percentage, *n* (%)							< 0.001
< 10%	14 (14.0)	28 (13.5)	224 (14.7)	54 (28.9)	21 (23.1)	32 (24.6)	
10–20%	67 (67.0)	156 (75.4)	1244 (81.7)	131 (70.0)	69 (75.8)	98 (75.4)	
> 20%	19 (19.0)	23 (11.1)	54 (3.6)	2 (1.1)	1 (1.1)	0 (0)	
Blastomere symmetry, *n* (%)							< 0.001
Even	74 (74.0)	150 (72.5)	1418 (93.2)	106 (56.7)	60 (65.9)	89 (68.5)	
Uneven	26 (26.0)	57 (27.5)	104 (6.8)	81 (43.3)	31 (34.1)	41 (31.5)	
Multinucleation or vacuoles, *n* (%)	1 (1.0)	2 (1.0)	23 (1.5)	2 (1.1)	1 (1.1)	0 (0)	0.889

Data are presented as median (Q25, Q75) or number (percentage)

*LH* luteinizing hormone, *E*_*2*_ estradiol, *P* progesterone, *IVF *in vitro fertilization, *ICSI* intracytoplasmic sperm injection

Pregnancy outcomes according to blastomere number are detailed in Table 3. The rate of live birth was 19.0% (95% CI 11.3–26.7%), 27.1% (95% CI 21.0–33.1%), 38.9% (95% CI 36.5–41.4%), 32.1% (95% CI 25.4–38.8%), 44.0% (95% CI 33.8–54.2%) and 53.8% (95% CI 45.3–62.4%) for the ≤ 6-cell, 7-cell, 8-cell, 9-cell, 10-cell and ≥ 11-cell groups, respectively. LBR varied significantly among groups (*P* < 0.001). Likewise, the positive hCG test and clinical pregnancy rates also rose significantly with increased blastomere number (both *P* < 0.001). Nonetheless, the difference was not detected in the proportion of either early or late miscarriage (*P* = 0.702 and 0.971, respectively).

**Table 3:** Pregnancy outcomes grouped by blastomere number

	≤ 6-cell (*n* = 100)	7-cell (*n* = 207)	8-cell (*n* = 1522)	9-cell (*n* = 187)	10-cell (*n* = 91)	≥ 11-cell (*n* = 130)	*P*-value
Positive hCG test, *n* (%)	29 (29.0)	84 (40.6)	872 (57.3)	89 (47.6)	57 (62.6)	88 (67.7)	< 0.001
Clinical pregnancy, *n* (%)	24 (24.0)	67 (32.4)	743 (48.8)	72 (38.5)	49 (53.8)	83 (63.8)	< 0.001
Miscarriage, *n/N* (%)	5/24 (20.8)	11/67 (16.4)	150/743 (20.2)	12/72 (16.7)	9/49 (18.4)	13/83 (15.7)	< 0.001
Early miscarriage, *n/N* (%)	4/24 (16.7)	7/67 (10.4)	111/743 (14.9)	7/72 (9.7)	6/49 (12.2)	9/83 (10.8)	0.702
Late miscarriage, *n/N* (%)	1/24 (4.2)	4/67 (6.0)	39/743 (5.2)	5/72 (6.9)	3/49 (6.1)	4/83 (4.8)	0.971
Live birth, *n* (%)	19 (19.0)	56 (27.1)	592 (38.9)	60 (32.1)	40 (44.0)	70 (53.8)	< 0.001

Data are presented as number (percentage)

*hCG* human chorionic gonadotropin

As shown in Table 4, after controlling for potential confounding factors, the ≤ 6-cell group was associated with lower LBR compared with the 8-cell group (aOR 0.50, 95% CI 0.29–0.86; *P* = 0.013). Conversely, the likelihood of live birth was significantly higher in patients transferred with 10-cell embryos (aOR 1.62, 95% CI 1.03–2.53; *P* = 0.035) and ≥ 11-cell embryos (aOR 2.14, 95% CI 1.47–3.11; *P* < 0.001). The odds of positive hCG test (aOR 0.67, 95% CI 0.49–0.93; *P* = 0.018) and clinical pregnancy (aOR 0.65, 95% CI 0.46–0.91; *P* = 0.012) were also decreased in the 7-cell group as compared with that in the reference group, while the difference in LBR (aOR 0.74, 95% CI 0.52–1.06; *P* = 0.106) failed to reach statistical significance. In the multiple regression model, miscarriage risk remained similar among blastomere number categories.

**Table 4:** Crude and adjusted odds ratios of pregnancy outcomes in blastomere number categories

	≤ 6-cell	7-cell	8-cell	9-cell	10-cell	≥ 11-cell
Positive hCG test						
Crude OR (95% CI)	0.30 (0.19–0.47)	0.51 (0.38–0.68)	Reference	0.68 (0.50–0.92)	1.25 (0.81–1.93)	1.56 (1.06–2.29)
*P*-value	< 0.001	< 0.001	-	0.012	0.320	0.023
Adjusted OR (95% CI)	0.43 (0.27–0.68)	0.67 (0.49–0.93)	Reference	0.84 (0.60–1.18)	1.65 (1.04–2.61)	1.77 (1.19–2.63)
*P*-value	< 0.001	0.018	-	0.322	0.032	0.005
Clinical pregnancy						
Crude OR (95% CI)	0.33 (0.21–0.53)	0.5 (0.37–0.68)	Reference	0.66 (0.48–0.90)	1.22 (0.80–1.87)	1.85 (1.28–2.69)
*P*-value	< 0.001	< 0.001	-	0.008	0.349	0.001
Adjusted OR (95% CI)	0.46 (0.28–0.76)	0.65 (0.46–0.91)	Reference	0.8 (0.56–1.13)	1.58 (1.02–2.46)	2.11 (1.44–3.10)
*P*-value	0.002	0.012	-	0.201	0.042	< 0.001
Miscarriage						
Crude OR (95% CI)	1.04 (0.38–2.83)	0.78 (0.40–1.51)	Reference	0.79 (0.41–1.51)	0.89 (0.42–1.88)	0.73 (0.40–1.35)
*P*-value	0.938	0.458	-	0.476	0.758	0.322
Adjusted OR (95% CI)	0.87 (0.27–2.86)	0.59 (0.29–1.20)	Reference	0.59 (0.29–1.20)	0.72 (0.34–1.54)	0.64 (0.33–1.24)
*P*-value	0.824	0.148	-	0.143	0.400	0.183
Live birth						
Crude OR (95% CI)	0.37 (0.22–0.61)	0.58 (0.42–0.81)	Reference	0.74 (0.54–1.03)	1.23 (0.80–1.89)	1.83 (1.28–2.62)
*P*-value	< 0.001	0.001	-	0.072	0.336	< 0.001
Adjusted OR (95% CI)	0.50 (0.29–0.86)	0.74 (0.52–1.06)	Reference	0.91 (0.63–1.32)	1.62 (1.03–2.53)	2.14 (1.47–3.11)
*P*-value	0.013	0.106	-	0.630	0.035	< 0.001

Adjusted for maternal age and BMI, infertility duration, infertility type, infertility diseases, IVF failure history, total gonadotropin dose, trigger day E_2_ level, P level and endometrial thickness, number of oocytes retrieved, fertilization method, fragmentation percentage, blastomere symmetry, and presence of multinucleation or vacuoles

*OR* odds ratio, *CI* confidence interval, *hCG* human chorionic gonadotropin

Regarding other embryo morphological characteristics, the details of covariate adjustment for live birth are presented in Table S1. The LBR of 10–20% (aOR 0.73, 95% CI 0.54–0.97; *P* = 0.028) and > 20% (aOR 0.41, 95% CI 0.23–0.73; *P* = 0.002) fragmentation was lower than that of < 10% fragmentation. Uneven cell size was associated with reduced LBR as compared with even size (aOR 0.49, 95% CI 0.35–0.69; *P* < 0.001). In addition, a decreased yet not significantly different LBR was observed in the presence of multinucleation or vacuoles (aOR 0.46, 95% CI 0.19–1.12; *P* = 0.088).

Descriptive statistics for the obstetric and neonatal outcomes are summarized in Table S2. Monozygotic twinning occurred in a total of 19 (0.8%) patients. No significant differences were found in all analyzed complications.

## Discussion

Over the past decade, there has been an increasing trend of extending culture from cleavage-stage embryos to blastocysts [18]. However, day 3 embryo transfer is still very common in many IVF centers [19, 20], due to its advantages of shorter in vitro culture duration, lower cancellation rate with no embryos, and greater number of embryos for freezing [21]. The results of this retrospective cohort study demonstrated a significant relationship between blastomere number and pregnancy outcomes in day 3 embryo transfer cycles. Compared with normally progressing embryos (8-cell), slow-cleaving embryos (≤ 6-cell) resulted in lower LBRs whereas higher LBRs were observed in fast-cleaving embryos (≥ 10-cell).

Our findings are in agreement with the general consensus on the decreased developmental competence of slow-cleaving day 3 embryos [6, 7]. In this regard, several possible reasons have been proposed with the application of time-lapse technology, including prolonged cell cycle, fragmentation leading to fewer surviving blastomeres, and developmental arrest or unexplained delays [14]. Slow-cleaving embryos were also reported to have higher aneuploidy rates and reduced likelihood to give rise to euploid blastocysts [12, 13]. As a result, the chromosomal abnormalities may cause implantation failure following embryo transfer.

To our knowledge, only limited studies have been conducted to investigate the impact of increased day 3 cell number on clinical outcomes of cleavage-stage embryo transfer cycles [6, 14, 15]. Based on the national SART CORS database, Racowsky et al*.* [6] found that there was a significant decline in LBR from embryos comprised of 8 cells to those with higher cell counts. Nevertheless, potential bias may have been introduced since the blastomere number was not recorded accurately and no confounders were controlled in the registry data, such as female age and infertility indications. Additionally, the inclusion of double embryo transfers made it hard to differentiate the morphological effects of independent embryos. In 2015, Kong et al*.* [14] contrarily showed that the LBR had an increasing trend with higher day 3 cell number after excluding embryos with abnormal division behaviors. This study was strengthened by the continuous monitoring with time-lapse imaging but similarly, no multivariate analyses were performed to confirm the results. To add further to the confusion, Zhao et al*.* [15] reported comparable LBR (60.0% *vs.* 59.90%; *P* = 0.98) but reduced miscarriage rate (4.3% *vs.* 13.5%; *P* = 0.04) in patients transferred with > 10-cell embryos compared to the 8-cell transfer group. However, all cycles were double rather than single embryo transfers and merely 75 cycles were involved in the > 10-cell group. Therefore, the statistical power may be insufficient to draw a robust conclusion.

The present study, aiming to overcome the drawbacks of previously published studies, re-analyzed the pregnancy outcomes following fast-cleaving embryo transfers. Our results, based on 2237 fresh single day 3 embryo transfer cycles, clearly demonstrated that embryos with ≥ 10 cells had similar miscarriage rate but significantly higher odds of live birth than 8-cell embryos.

The reason why increased blastomere number associates with improved LBR remains unclear. It is speculated that the difference in blastocyst formation and morphological quality may play a role. Indeed, while fast-cleaving embryo on day 3 was reported in some studies to have comparable blastocyst formation rate to 8-cell embryo [8, 9, 11, 13], other researchers showed a statistically significant elevation [12, 14]. Moreover, regression analysis by Shapiro et al*.* [8] indicated that the probability of blastocyst expansion increased by 7.6% *per* day 3 cell (*R*^2^ = 0.847; *P* = 0.009), and embryo with ≥ 9 cells were more likely to develop to expanded blastocyst compared with 8-cell embryo (74% *vs.* 49%; *P* = 0.022). Likewise, a retrospective study by Luna et al*.* [10] suggested that faster developing embryos (≥ 10-cell) had a significantly greater likelihood to become high-quality blastocysts with grade 4AA or 5AA than intermediate-cleaving embryos. Therefore, increased day 3 cell number may be related to higher developmental competence and consequently lead to higher LBR.

However, it could be argued that chromosomal abnormalities may occur more frequently in fast-cleaving embryos. Among the supporting evidences [12, 22, 23], the latest work, on a basis of 1915 embryo biopsies, demonstrated that a number of day 3 blastomeres > 9 was significantly correlated with increased aneuploidy rates (OR 1.39; *P* = 0.029) [12]. Nonetheless, these results were contradicted with Moayeri et al*.* [24] showing that cellular fragmentation, instead of cell number, acted as a sensitive proxy for predicting chromosomally normal embryos. In another retrospective study, Zhao et al*.* [15] also found that > 10-cell derived blastocysts had similar aneuploidy rates to 8-cell derived blastocysts (55.6% *vs.* 55.9%). More recently, Pons et al*.* [13] analyzed the PGT-A data of 4028 embryos in total and further confirmed that the ploidy of > 11-cell embryos was comparable with that of 8-cell embryos at the blastocyst stage (OR 1.20, 95% CI 0.92–1.56). Hence, the implantation potential of fast-cleaving embryos may not be compromised by its chromosomal status.

In addition to pregnancy outcomes, our study further demonstrated that the obstetrical and perinatal complications were largely unaffected by blastomere number on day 3. This observation is to some extent discordant with a previous study showing that blastocysts derived from ≥ 10-cell embryos had significantly lower incidences of low birthweight (0.9% *vs.* 5.3%; *P* = 0.04) and preterm birth (2.6% *vs.* 8.1%; *P* = 0.04) than the 8-cell group [25]. While the authors attributed it to the continuously rapid developmental speed of fast-cleaving embryos, this hypothesis, as they acknowledged, requires more researches for validation due to the borderline significance. Instead, the present work found that the percentage of males tended to be higher in the ≥ 11-cell embryo group, further supporting the potential influence of embryo sex on embryo development [26, 27].

The major strength of our study is the large cohort size; to date, this is the largest study concerning the effect of blastomere number on pregnancy outcomes after single day 3 embryo transfers. During the study period, all laboratory and clinical practices remained consistent in our center, where embryo grading was conducted by two highly experienced embryologists to reduce inter-observer variation. Furthermore, multiple regression models were established to control for a variety of confounding factors, including patient characteristics, cycle features as well as embryo morphological parameters.

Certain limitations should be acknowledged of the present work. On the one hand, it was a retrospective cohort study with inherent bias. The number of cycles in each group was not equally distributed and some baseline variables were not comparable across groups. However, this circumstance is also rational as 8-cell embryos could be preferentially selected for transfer in current practice, while randomized controlled trials are out of the question for ethical principles. On the other hand, maternal and neonatal outcomes were mainly self-reported by patients. While telephone surveys were conducted by our specially trained nurses, the reliability of these information may still be compromised and medical records should be reviewed for data collection in future studies. In addition, given the low incidence of these complications, the present study could be underpowered to detect significant differences, and further prospective cohorts with larger sample size and adequate statistical power are required for more assertive evidence.

In conclusion, our study showed that increased blastomere number, an important indicator for cell cycle progression, was associated with higher LBR in fresh single day 3 embryo transfer cycles. This finding questions the consensus on the reduced developmental potential of fast-cleaving embryos, which should be included and even prioritized for transfer based on our data. Further prospective studies with larger sample size are warranted for confirmation.

## Supplementary Information

**Additional file 1: Table S1.** Results of multiple regression analysis for the association of embryo morphological features with live birth rate.

**Additional file 2: Table S2.** Obstetric and neonatal outcomes grouped by blastomere number.

## Data Availability

The datasets used and/or analyzed during the current study are available from the corresponding author on reasonable request.

## Abbreviations

aOR: Adjusted odds ratio
BMI: Body mass index
CI: Confidence interval
E_2_: Estradiol
ESHRE: European Society of Human Reproduction and Embryology
FSH: Follicle-stimulating hormone
GEE: Generalized estimating equation
hCG: Human chorionic gonadotrophin
ICSI: Intracytoplasmic sperm injection
IVF: In vitro fertilization
LBR: Live birth rate
LH: Luteinizing hormone
P: Progesterone
PGT-A: Preimplantation genetic testing for aneuploidy
SART CORS: Society for Assisted Reproductive Technology Clinic Outcome Reporting System
