# Supplementary material for: Increased blastomere number is associated with higher live birth rate in day 3 embryo transfer
Source: BMC Pregnancy Childbirth. 2022 Mar 11;22:198. doi: 10.1186/s12884-022-04521-5 (PMC8917733; doi:10.1186/s12884-022-04521-5)
Supplement: Supplementary file 1 — Additional file 1: Table S1. Results of multiple regression analysis for the association of embryo morphological features with live birth rate. [file 12884_2022_4521_MOESM1_ESM.docx]

**Table S1.** Results of multiple regression analysis for the association of embryo morphological features with live birth rate.

|  | Adjusted odds ratio (95% confidence interval) | *P*-value |
| --- | --- | --- |
| Fragmentation percentage |  |  |
| <10% | Reference |  |
| 10–20% | 0.73 (0.54–0.97) | 0.028 |
| >20% | 0.41 (0.23–0.73) | 0.002 |
| Blastomere symmetry |  |  |
| Even | Reference |  |
| Uneven | 0.49 (0.35–0.69) | <0.001 |
| Multinucleation or vacuoles |  |  |
| No | Reference |  |
| Yes | 0.46 (0.19–1.12) | 0.088 |
