# Supplementary material for: Increased blastomere number is associated with higher live birth rate in day 3 embryo transfer
Source: BMC Pregnancy Childbirth. 2022 Mar 11;22:198. doi: 10.1186/s12884-022-04521-5 (PMC8917733; doi:10.1186/s12884-022-04521-5)
Supplement: Supplementary file 2 — Additional file 2: Table S2. Obstetric and neonatal outcomes grouped by blastomere number. [file 12884_2022_4521_MOESM2_ESM.docx]

**Table S2.** Obstetric and neonatal outcomes grouped by blastomere number.

|  | ≤6-cell | 7-cell | 8-cell | 9-cell | 10-cell | ≥11-cell | *P*-value |
| --- | --- | --- | --- | --- | --- | --- | --- |
| ***Obstetric outcomes*** | ***n* = 24** | ***n* = 67** | ***n* = 743** | ***n* = 72** | ***n* = 49** | ***n* = 83** |  |
| Hypertensive disorders of pregnancy, *n* (%) | 1 (4.2) | 3 (4.5) | 20 (2.7) | 0 (0) | 1 (2.0) | 3 (3.6) | 0.460 |
| Gestational diabetes mellitus, *n* (%) | 1 (4.2) | 6 (9.0) | 53 (7.1) | 5 (6.9) | 4 (8.2) | 9 (10.8) | 0.817 |
| Intrahepatic cholestasis of pregnancy, *n* (%) | 0 (0) | 0 (0) | 2 (0.3) | 0 (0) | 0 (0) | 1 (1.2) | 0.634 |
| Placenta previa, *n* (%) | 1 (4.2) | 1 (1.5) | 7 (0.9) | 1 (1.4) | 0 (0) | 0 (0) | 0.365 |
| ***Neonatal outcomes*** | ***n* = 20** | ***n* = 58** | ***n* = 604** | ***n* = 60** | ***n* = 41** | ***n* = 73** |  |
| Male/female ratio | 12/8 (1.50:1) | 25/33 (0.76:1) | 320/284 (1.13:1) | 29/31 (0.94:1) | 20/21 (0.95:1) | 49/24 (2.04:1) | 0.097 |
| Gestational age (weeks) | 38.24±2.27 | 38.84±1.45 | 38.72±1.59 | 38.61±2.05 | 38.41±2.36 | 38.70±1.38 | 0.628 |
| Preterm birth, *n* (%) | 5 (25) | 5 (8.6) | 57 (9.4) | 7 (11.7) | 8 (19.5) | 6 (8.2) | 0.102 |
| Very preterm birth, *n* (%) | 0 (0) | 0 (0) | 4 (0.7) | 1 (1.7) | 1 (2.4) | 0 (0) | 0.412 |
| Post-term birth, *n* (%) | 0 (0) | 0 (0) | 0 (0) | 1 (1.7) | 0 (0) | 0 (0) | 0.209 |
| Birthweight (g) | 3222.5±689.3 | 3221.2±423.3 | 3221.5±508.7 | 3231.7±687.7 | 3262.2±539.5 | 3312.7±529.9 | 0.831 |
| Low birthweight, *n* (%) | 4 (20) | 3 (5.2) | 42 (7.0) | 4 (6.7) | 3 (7.3) | 4 (5.5) | 0.406 |
| Very low birthweight, *n* (%) | 0 (0) | 0 (0) | 3 (0.5) | 1 (1.7) | 1 (2.4) | 0 (0) | 0.289 |
| Macrosomia, *n* (%) | 2 (10) | 3 (5.2) | 35 (5.8) | 6 (10) | 1 (2.4) | 9 (12.3) | 0.171 |
| Major congenital malformations, *n* (%) | 1 (5) | 0 (0) | 6 (1.0) | 2 (3.3) | 0 (0) | 2 (2.7) | 0.215 |
